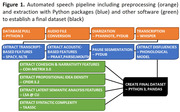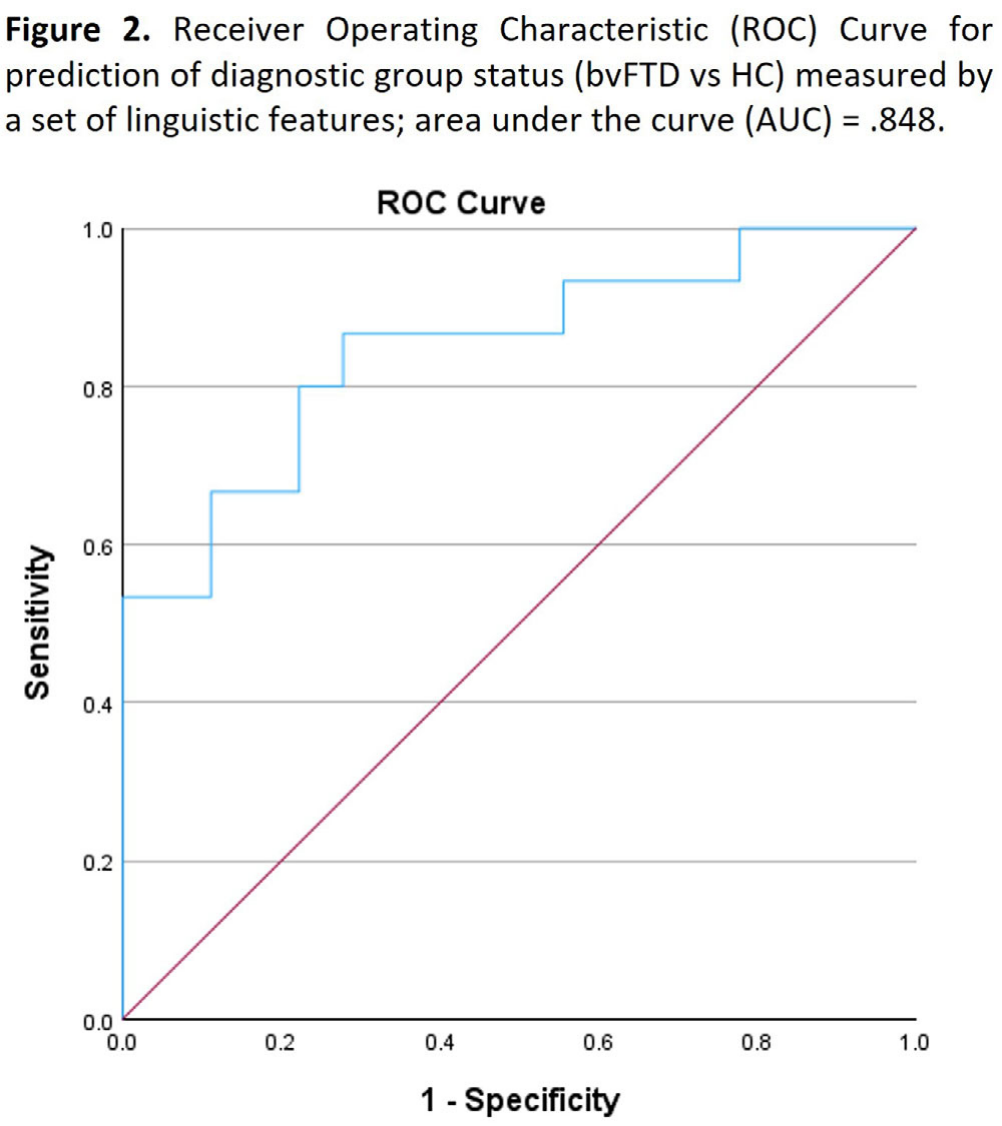# Remote Naturalistic Speech Analysis as a Tool to Classify Behavioral Variant Frontotemporal Dementia

**DOI:** 10.1002/alz.091734

**Published:** 2025-01-03

**Authors:** Jet M. J. Vonk, Brittany T. Morin, Sreya Dhanam, Annie L. Clark, J. Clayton Young, Jack C. Taylor, David Rosado‐Rolon, Hilary W. Heuer, Leah K. Forsberg, Brad F. Boeve, Howard J. Rosen, Maria Luisa Gorno Tempini, Adam L. Boxer, Adam M. Staffaroni

**Affiliations:** ^1^ University of California San Francisco (UCSF), San Francisco, CA USA; ^2^ Mayo Clinic, Rochester, MN USA; ^3^ University of California San Francisco, San Francisco, CA USA; ^4^ Memory and Aging Center, Weill Institute for Neurosciences, University of California, San Francisco, San Francisco, CA USA

## Abstract

**Background:**

Frontotemporal dementia (FTD) is a diverse spectrum of neurodegenerative disorders impacting the frontal and temporal lobes, primarily manifesting as behavioral and/or language issues. Accurate diagnosis is crucial for effective management, potential treatment, and care planning. While speech analysis has shown promise in detecting cognitive markers of Alzheimer’s disease (AD), its exploration in FTD is limited. This study examined if linguistic features from remote, app‐based speech collection can differentiate healthy controls (HC) from individuals with behavioral variant FTD (bvFTD), the most common FTD variant.

**Method:**

Participants included 15 bvFTD (mean age = 64.3, SD = 9.1 years) and 19 HC (mean = 55.8, SD = 14.2) from the ALLFTD Consortium. Participants were prompted to talk freely about a happy memory; audio recordings were collected with the ALLFTD Mobile App, transcribed using OpenAI’s large Whisper model, and manually quality‐checked. We extracted 98 linguistic features using our in‐house pipeline (Fig 1). Features were selected for classification analysis if they differed between groups based on a MANCOVA adjusted for age, sex/gender, and education, with feature reduction in case of collinearity. We then determined if these features could classify groups using multi‐variable logistic regression models, and subsequently calculated its ROC‐AUC score to measure sensitivity/specificity.

**Result:**

Based on the MANCOVA, three features were selected for classification analysis: average seconds of non‐speech (i.e., silence), ratio of number of complex nominal clauses to total clauses, and number of restarts (i.e., disfluency). The logistic model showed that this set could distinguish between groups (Omnibus p = .001; Nagelkerke R^2^ = .499), and their saved prediction probabilities indicated a ROC‐AUC score of 84.8% accuracy (Fig 2).

**Conclusion:**

Leveraging a flexible speech capture app with remote accessibility and state‐of‐the‐art automated speech analysis, we identified linguistic features that can distinguish HC from bvFTD. This approach presents a promising screening tool for detecting bvFTD, offering quick, easily administered assessments with the potential for scalable implementation outside of clinical settings. Next steps are to investigate linguistic changes in presymptomatic mutation carriers and compare speech profiles between FTD and AD. These results hold meaningful implications for addressing current challenges in FTD therapeutics, potentially offering sensitive outcome measures for clinical trials and future tools for personalized cognitive assessment and long‐term monitoring.